# Effectiveness of a Census-Based Management Information System for Guiding Polio Eradication and Routine Immunization Activities: Evidence from the CORE Group Polio Project in Uttar Pradesh, India

**DOI:** 10.4269/ajtmh.18-0935

**Published:** 2019-10

**Authors:** Manojkumar Choudhary, Henry B. Perry, Roma Solomon

**Affiliations:** 1 CORE Group Polio Project/India, Gurgaon, India;; 2 Department of International Health, Johns Hopkins Bloomberg School of Public Health, Baltimore, Maryland

## Abstract

Census-based management information systems (CB-MISs) bring strength and power to public health programs by providing current information about everyone in the population covered by the program. Such a system has been developed by the CORE Group Polio Project (CGPP) in India. This article assesses the effectiveness of the CGPP CB-MIS in the management of social and behavioral change interventions. It also assesses the feasibility of the CB-MIS for vital events registration. We describe the procedures of the CB-MIS and measure the outcomes of the CGPP by observing the trends of vaccination coverage in CGPP catchment areas over time. We also compute vital statistics from births and deaths registered through the CGPP CB-MIS and compare them with the estimates from the Civil Registration System of India using statistics from India’s Sample Registration System in Uttar Pradesh as the “gold standard.” The CB-MIS has helped the CGPP to manage its social and behavior change communication interventions effectively, and it has contributed to the increase in polio vaccine coverage facilitated by the CGPP. We also estimate that the CGPP’s CB-MIS has registered 86% of births and 98% of infant deaths, a much higher level of registration than has been achieved by the Civil Registration System for the entire state of Uttar Pradesh. The CB-MIS has helped to make it possible for community-based health workers to make behavioral diagnoses of barriers to immunization and to overcome them. The CB-MIS also provides a robust platform for community-based health workers to register vital events.

## INTRODUCTION

A census-based management information system (CB-MIS) can bring strength and power to public health programs by providing current information about everyone in the population covered by the program. A CB-MIS can contribute to the effective planning and execution of any project or program that aims to engage with everyone in the population. Various types of CB-MISs have been used for different public health purposes, such as demographic surveillance, disease surveillance, vaccine safety assessments, epidemiological research, and clinical trials.^1–6^

Few studies in the peer-reviewed literature assess the contributions that a CB-MIS can make to the planning, monitoring, and evaluation of social and behavior change communication (SBCC) interventions. This article describes the effectiveness of the CORE Group Polio Project (CGPP) program to improve polio and routine immunization (RI) coverage in underserved populations, the contribution of its CB-MIS to achieving these results, the strength of the vital events registration system embodied within the CB-MIS, and the potential of this CB-MIS for contributing to the effectiveness of other public health programs in similar environments.

## THE CGPP IN INDIA AND ITS SOCIAL AND BEHAVIORAL CHANGE INTERVENTIONS

The CORE Group is an association of nongovernmental organizations that engage with communities to help them improve their health.^7^ With financial assistance from the U.S. Agency for International Development and the Bill & Melinda Gates Foundation, the CGPP currently supports polio eradication initiatives and related activities in high-risk geographic areas of eight countries (Afghanistan, Ethiopia, India, Kenya, Nigeria, Somalia, South Sudan, and Uganda).^8^ In India, the CGPP works in high-priority areas of 12 districts in the state of Uttar Pradesh with a total population of approximately three million people. Since 2018, the CGPP in India also initiated a block-level intervention in two blocks of Nuh district of Haryana state. Two other articles in this series provide further descriptions of the CGPP work in India.^9,10^

The CGPP is a member of the Social Mobilization Network (SMNet) in India, along with the United Nations Children’s Fund (UNICEF), Rotary International, the government of India (GoI), and the WHO’s National Polio Surveillance Project. Since its formation in 2003,^11^ the SMNet has supported polio eradication through the following efforts: identifying high-risk areas and working with underserved communities in planning, implementing, and monitoring social mobilization and other immunization-related activities. The activities of the SMNet are carried out by community-based health workers called community mobilization coordinators (CMCs) at the community level, who are supervised by block mobilization coordinators (BMCs),† who are in turn supervised by district mobilization coordinators.

The CGPP/India has its own CB-MIS which contains information on all households and the individuals living in each household in the CGPP catchment areas. This CB-MIS tracks inputs, outputs, processes, and outcomes of its SBCC interventions. The CMCs are each responsible for 500 or so households and maintain registers about each of them, including the current vaccination status of every child. Although this information has been used since the CGPP began its work in India in 1999, the CB-MIS has become more comprehensive and robust over time.

The CGPP reaches every household and tracks the immunization status of children younger than 5 years by direct personal communication with families. When resistance is encountered, the CGPP facilitates engagement with informal and formal community leaders.^11^ A network of 1,100 CMCs conducts social mobilization activities for social and behavior change related to polio vaccination and RI. Supplemental Appendix I contains details of the CGPP’s SBCC activities for polio Supplementary Immunization Activities (SIAs) and the CGPP’s activities for RIs, including routine polio immunization.

## DESCRIPTION OF THE CB-MIS

The CB-MIS of the CGPP/India uses prospectively and retrospectively collected information for planning, implementation, monitoring, and evaluation of its SBCC activities. Key input, process, output, and outcome indicators of the project are tracked by internal and external assessments. In addition to routinely collecting data, the CGPP also conducts specific investigations using both quantitative and qualitative techniques, such as lot quality assurance sampling (LQAS) surveys and barrier analysis (to identify barriers families face in adopting healthy behaviors). The CB-MIS aims to support program implementation teams in the effective management (planning, implementation, monitoring, supervision, and evaluation) of SBCC interventions at various levels—from the household to the community to the block and to the district levels—for polio eradication in the CGPP catchment areas.

**Figure 1.**
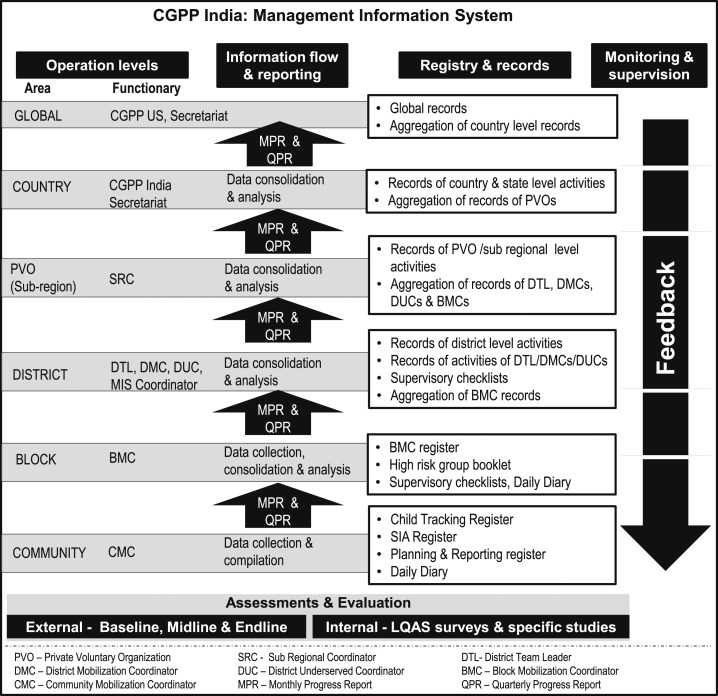
The structure and function of the census-based management information system of the CGPP/India.

Figure 1 describes the various registries, tools, and records that make up the CB-MIS; the various levels and stages of data collection, compilation, analysis, and reporting at each operational level; and the flow of information between levels of operation. The Assessment and Evaluation section (at the bottom of Figure 1) lists the various data sources that the CGPP uses for managing the system.

The CB-MIS generates real-time information from the grassroots. All information is collected and consolidated according to a common protocol developed by the CGPP, which uses the same indicators as the SMNet and the RI system. At the community level, CMCs maintain a Child Tracking Register, an SIA Register, and a Planning and Reporting Register. The CB-MIS provides a structure for collecting, processing, and disaggregating information for tracking individual children and for planning SIAs and RI activities. The Supplemental Appendix provides additional information about the registers used in the CB-MIS.

## METHODS

### Data.

This article uses data from October 2014 to September 2017 that were originally collected for the CB-MIS, including denominator populations, immunization status of children, and registration of pregnant women, newborns (births), and infant deaths. We also use 11 years of SIA coverage data (from 2007 to 2017) for areas adjacent to CGPP catchment areas that were compiled through the CB-MIS of the CGPP. These data were obtained as follows. After every SIA, BMCs collect block-level statistics regarding campaign coverage. The block-level data include aggregated information from both the CMC and non-CMC areas. Block mobilization coordinators compute statistics for non-CMC areas by subtracting CMC area data from the block-level data. In addition, vital statistics reported through the Civil Registration System and Sample Registration System (SRS) Bulletin of India were used for comparative analyses.^12–14^ We also use findings of a data validation study that was conducted by CGPP/India in 2014 to ensure completeness and accuracy of reported data for newborn registration and routine vaccination coverage. In this data validation exercise, which used a 30-cluster sample (with 10 households per cluster), selected CGPP staff visited households to collect information.

### Analysis.

We assessed the effectiveness of the CGPP program by using data from the CB-MIS and observing the trends over time. The following indicators were calculated:

$$\text{Percentage}\;\text{of}\;\text{missed}\;\text{houses}\;\text{during}\;\text{each}\;\text{SIA}:\;\frac{\text{Number}\;\text{of}\;\text{missed}\;\text{houses}\;\text{during}\;\text{the}\;\text{SIA}}{\text{Number}\;\text{of}\;\text{targeted}\;\text{houses}}\;\times \;100$$

$$\text{Percentage}\;\text{of}\;\text{vaccinated}\;\text{children}:\;\frac{\text{Number}\;\text{of}\text{children}\;\text{vaccinated}\;\text{with}\;\text{a}\;\text{particular}\;\text{vaccine}}{\text{Number}\;\text{of}\;\text{children}\;\text{eligible}\;\text{for}\;\text{that}\;\text{particular}\;\text{vaccine}}\;\times \;100$$

We assess the value of the CB-MIS by describing its utility in planning, implementing, and evaluating CGPP activities.

The SRS is a vital events registration system maintained in sample districts by the GoI, which is widely considered to be the “gold standard” of vital events registration in India. The SRS is a dual registration system obtained by one worker (resident part-time enumerator of an SRS site–sampled area) who visits every house monthly to register new vital events since the previous visit (continuous enumeration) and by a different worker (full-time supervisor, SRS) who visits every house every 6 months to register vital events that occurred during the previous 6 months (retrospective half-yearly survey). Vital events missed by one system are likely to be identified by the other system.^15^ This intensive system provides accurate vital statistics at the country level and for the bigger states with more than 10 million population,^16^ but it is not feasible to implement such a system in all the blocks or districts of the country.

To assess the accuracy of the CB-MIS in registering vital events, we calculated the pregnancy registration rate, crude birth rate (CBR), and infant mortality rate (IMR) with data from the CB-MIS. We computed the level of vital events registration for CGPP work areas. We define the level of registration by dividing the number of vital events (i.e., births and infant deaths) in the CGPP work areas that were recorded by either the CGPP CB-MIS or the Civil Registration System by the number predicted by the SRS using locally derived SRS statistics, and then multiplying this by 100 to obtain a percentage. The levels of vital events registration during the period from October 2014 to September 2017 in the CGPP CB-MIS were compared with those of the Civil Registration System.^14^ We then compared these rates of pregnancy, birth, and infant mortality produced by these two different systems of vital events registration.

We compared the data from the validation exercise in 2014 with the information in the CMC/BMC registers to assess the completeness and accuracy of the CGPP CB-MIS. Data accuracy in reporting of vaccination coverage was measured as a ratio between the vaccination coverage reported through the CB-MIS (CMC registers) and the vaccination coverage estimated through the household survey. The first systematic attempt to assess the quality of reported data was in 2014. Then, the CGPP conducted such exercises at least once a year.

All the primary vaccines used in RI (birth dose of oral polio vaccine [OPV0]; Bacille Calmette-Guerin [BCG]; diphtheria, pertussis and tetanus first dose [DPT1], DPT2, DPT3; OPV first dose [OPV1], OPV2, OPV3; hepatitis B birth dose [hepatitis B0]; hepatitis B first dose [hepatitis B1], hepatitis B2, hepatitis B3, and measles) were assessed for data accuracy using the following formula:

$$\frac{\text{Vaccination}\;\text{coverage}\;\left(\text{for}\;\text{a}\;\text{given}\;\text{vaccine}\;\text{dose}\right)\;\text{reported}\;\text{through}\;\text{the}\;\text{CB}-\text{MIS}}{\text{Vaccination}\;\text{coverage}\;\left(\text{for}\;\text{a}\;\text{given}\;\text{vaccine}\;\text{dose}\right)\;\text{estimated}\;\text{through}\;\text{household}\;\text{survey}}\times 100$$

Accuracy ratio of 100% was defined as “perfect quality of reporting,” and observed accuracy ratios of above and below 100% were labeled as over- and underreporting, respectively. Furthermore, computed RI coverage accuracy ratios were divided into five categories: 1) perfect-quality reporting (accuracy ratio of 100%), 2) good-quality reporting (accuracy ratio from 90% to 99% or 101% to 109%), 3) moderate-quality reporting (accuracy ratio from 70% to 89% or 110% to 129%), 4) poor-quality reporting (accuracy ratio from 50% to 69% or 130% to 149%), and 5) extremely poor quality of reporting (accuracy ratio either below 50% or above 150%).

## RESULTS

### Use of the CB-MIS to assess participation in supplemental immunization activities.

**Figure 2.**
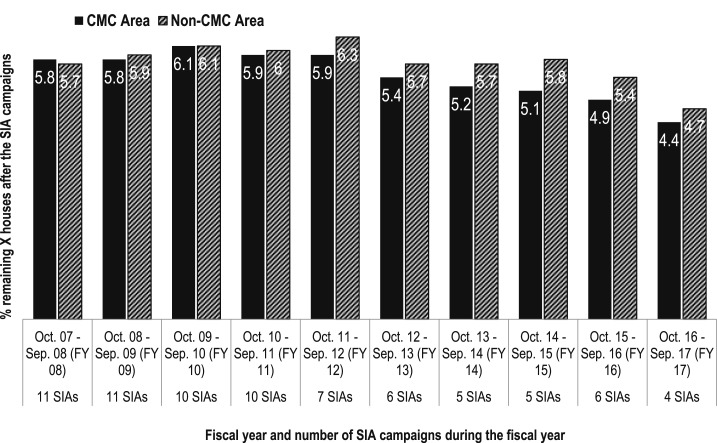
Percentage of houses missed during the supplemental immunization activity campaigns in the CGPP work areas in Uttar Pradesh, India, 2007–2017.

In Uttar Pradesh, the outcome of a given polio SIA is assessed by calculating the percentage of eligible households that did not participate in the SIA. The high-risk areas where the CGPP implements community-level SBCC activities through the CMCs have maintained a slightly lower percentage of missed houses compared with the non-CMC area (Figure 2). Although the difference in the percentage of “*X*” households between the CMC and non-CMC areas is modest, it is important to keep in mind that the CMC areas contain high-risk and difficult-to-reach households, have pockets of resistance to SIAs, or have low levels of immunization coverage. Therefore, achieving a level of performance in the CMC area that is comparable to that of the non-CMC areas is a major achievement.

**Figure 3.**
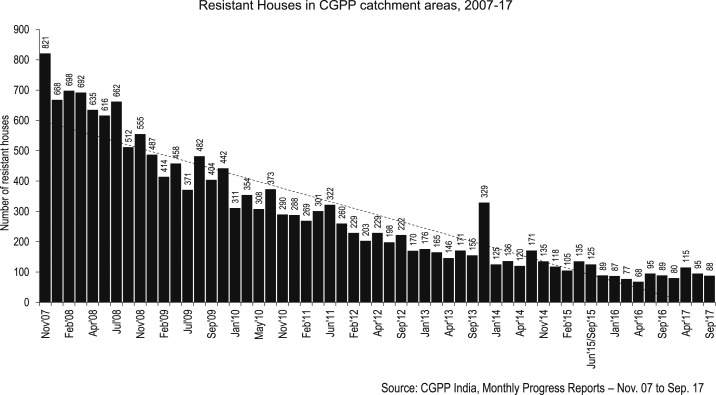
Number of houses missed in supplemental immunization activities due to resistance in the catchment areas of the CGPP in India, 2007–2017.

In 2006, SIA operations in Uttar Pradesh began to involve local influencers who were willing to help reduce resistance against polio vaccination. Figure 3 presents the trend in the number of houses missed during SIAs due to resistance in the catchment areas of the CGPP in India. It is evident that the number of resistant households has significantly decreased from 821 in November 2007 to about 88 in September 2017.

### Use of the CB-MIS to monitor immunization coverage.

**Figure 4.**
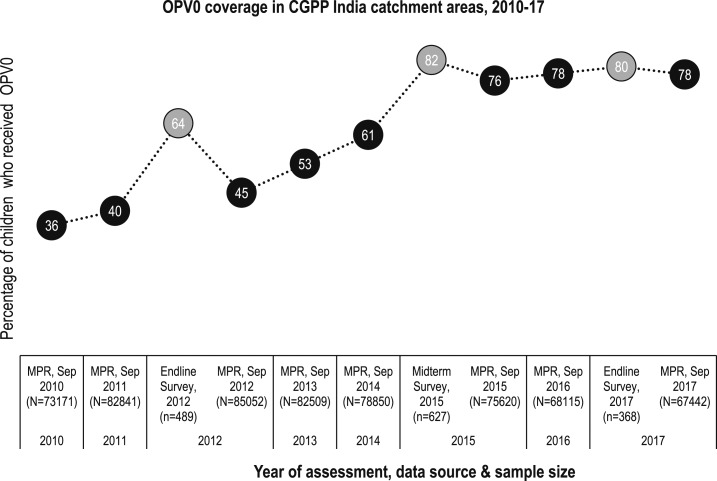
OPV0 (birth dose) coverage among children aged 12–23 months in the catchment areas of the CGPP in India, 2010–2017. Note: This graph uses data from two different sources of the census-based management information system. The black spheres represent OPV0 coverage reported through the monthly progress reports (MPR) of the CGPP, whereas the gray spheres show the coverage observed in baseline, midterm, and endline evaluations (based on independent household surveys).

**Figure 5.**
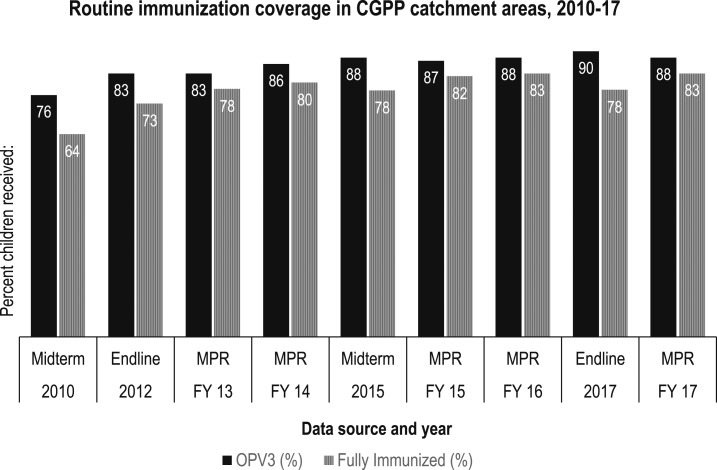
OPV3 and full immunization coverage (receipt of BCG, three doses of OPV, three doses of DPT/pentavalent vaccine, and measles vaccine) among children aged 12–23 months in the catchment areas of the CGPP/India, 2010–2017. Note: MPR = monthly progress report; midterm and endline refer to independent household surveys conducted at the specified points in time.

In the CGPP catchment areas, the birth dose coverage of OPV (i.e., the percentage of children who received their first dose of OPV within the first 15 days after birth) more than doubled from 36% in 2010 to 78% in 2017. The OPV birth dose, also known as OPV0, is given to newborns younger than 15 days. Both the demand- and supply-side factors (including the SBCC interventions of the CGPP) contributed in the increase of OPV0 coverage. The OPV3 coverage provided through RI has been maintained at above 80%, and full immunization coverage increased from 48% in 2008 to 78% in 2017 (Figures 4 and 5).

In the past 15 years, RI coverage in India has increased steadily, although the rate of increase has varied by states and districts. National-level full immunization coverage had increased from 44% in 2005–06 to 62% in 2015–16. In Uttar Pradesh, about 51% of children aged 12–23 months were fully immunized in 2015–16, an increase of 28 percentage points from 2005 to 2006.^17^ Increased vaccination coverage is a result of continuous efforts of the GoI to improve the vaccination services. The GoI has taken various steps and adopted the learnings of the national polio program for RI to accelerate the increase in vaccination coverage. For instance, in December 2014, a special immunization drive, *Mission Indradhanush* (MI), was launched to strengthen health systems for addressing equity issues in access to immunization. Furthermore, in 2017, the GoI has also introduced “Intensified MI,” strategized to cover all left-outs and dropouts in select districts and urban areas with low RI coverage and achieve the target of more than 90% full immunization coverage in a specific time-frame.^18^

Compared with the national- and state-level data on “percent fully immunized children,” CGPP/India reports higher (78%) vaccination coverage. Increases in vaccination coverage in the CGPP catchment areas were made possible using local data of the CB-MIS to target local immunization activities.^11,19–21^

### Use of the CB-MIS to register pregnant women, births, and infant deaths.

The CB-MIS provides information about the target population for each CMC catchment area (number of households and number of children younger than 5 years) and newly identified pregnant women, births, and deaths during the first year of life (Table 1). This information is used to calculate vaccination coverage rates, pregnancy registration rates, birth rates, and IMRs. The total population for the CGPP catchment areas is computed by multiplying the average family size (5.93 family members per household according to the 2011 government census of Uttar Pradesh)^22^ by the total number of households. The decline in the population of the CGPP catchment area is a result of the gradual withdrawal of the CGPP from 200 communities (a decrease from 1,300 to 1,100 communities between 2013 and 2017).

**Table 1:** Number of pregnancies, births, and infant deaths registered by CMCs, October 2012–September 2017

Number of vital events and population covered by CMCs	FY 13 (October 12–September 13)	FY 14 (October 13–September 14)	FY 15 (October 14–September 15)	FY 16 (October 15–September 16)	FY 17 (October 16–September 17)	October 2014–September 2017
Number of pregnant women registered by CMCs	NA	NA	70,208	70,594	70,931	211,733
Number of live births registered by CMCs	80,845	77,527	73,518	66,822	65,662	206,002
Number of infant deaths registered by CMCs	3,155	3,271	3,069	2,879	2,750	8,698
Number of households covered by CMCs	570,843	561,088	544,380	502,485	500,640	–
Number of children younger than 5 years in CMC areas	440,901	426,167	430,031	390,506	375,909	–
Estimated total population of CMC areas*	3,385,097	3,327,254	3,228,171	2,979,736	2,968,797	–

CMC = community mobilization coordinators; FY = fiscal year of the CGPP; NA = not available.

* Total population is estimated as: total number of households multiplied by the average family size of 5.93.

Table 2 shows the pregnancy registration rates, CBRs, and IMRs in the CGPP areas as computed from data in the CB-MIS for the years 2014–2017. Table 3 compares the rates for these 3 years combined with those from the SRS statistics of the entire state of Uttar Pradesh. The IMR computed by the CGPP CB-MIS (42) is virtually the same as the SRS IMR of 43. The CB-MIS CBR (22.4) is somewhat less than the SRS CBR (26.2).

**Table 2:** Estimated pregnancy registration rate, CBR, and IMR in the CGPP/India catchment areas using the community-based management information system data, October 2012–September 2017

Rates	FY 13 (October 12–September 13)	FY 14 (October 13–September 14)	FY 15 (October 14–September 15)	FY 16 (October 15–September 16)	FY 17 (October 16–September. 17)	October 2014–September 2017
Pregnancy registration rate (per 1,000 population)	NA	NA	21.7	23.7	23.9	23.1
CBR (per 1,000 population)	23.9	23.3	22.8	22.4	22.1	22.4
IMR (per 1,000 live births)	39.0	42.2	41.7	43.1	41.9	42.2

CBR = crude birth rate; IMR = infant mortality rate; NA = not available.

**Table 3:** Pregnancy, birth, and mortality rates obtained from vital events recorded in the census-based management information system for the CGPP catchment areas and from the SRS for Uttar Pradesh

Rates	CB-MIS (CGPP area) (October 2014–September 2017)		SRS (Uttar Pradesh), 2016*	
	Estimate	95% CI	Estimate	95% CI
Pregnancy registration rate (per 1,000 population)	23.1	22.9–23.2	NA	NA
Crude birth rate (per 1,000 population)	22.4	22.3–22.6	26.2	25.2–27.2
Infant mortality rate (per 1,000 live births)	42.2	40.7–43.7	43	39–48

CB-MIS = census-based management information system; CGPP = CORE Group Polio Project; SRS = Sample Registration System.

* Source of SRS data: SRS Bulletin, September 2017.^12^

Table 4 contains information on the number of births and deaths registered in the CGPP work areas and in Uttar Pradesh state (Table 4). The data for Uttar Pradesh are from the Civil Registration System, which registers vital events for the entire population. Similar data from the GoI’s SRS for the entire state of Uttar Pradesh are also presented. The level of registration, computed as described in the Methods section, is 86% for births and 98% for infant deaths, considerably better than the level of registration for the Civil Registration System in Uttar Pradesh (67% and 9%, respectively).

**Table 4:** Level of birth and death registration of CGPP community-based management information system and the civil registration system in Uttar Pradesh

Information	CGPP CB-MIS, October 2014–September 2017 (for CGPP catchment areas)		Civil Registration System, Uttar Pradesh, 2015 (for the entire state)	
	Data source	Values	Data source	Values
Total population	CB-MIS registries	3,058,901	Vital statistics of India, 2015^24^	215,835,000
Number of registered births		68,667		3,881,295
Number of expected births*	–	80,143	–	5,762,795
Estimated completeness of birth registration (using SRS data as the gold standard)†	–	85.7%	–	67.4%
Number of registered infant deaths	CB-MIS registries	2,899	Vital statistics of India, 2015^24^	15,410
Expected number of infant deaths‡	–	2,953	–	166,896
Estimated completeness of infant death registration (using SRS data as the gold standard)§	–	98.2%	–	9.2%

CB-MIS = census-based management information system; CBR = crude birth rate; CGPP = CORE Group Polio Project; IMR = infant mortality rate; SRS = Sample Registration System; data for the CORE Group Polio Project CB-MIS are averages for the three fiscal years (FY 15, FY 16, and FY 17).

* Expected number of births=Total population × CBR/1,000 (and CBR is assumed to be 26.2 for the state of Uttar Pradesh, based on SRS data^12^).

† $\text{Estimated}\;\text{completeness}\;\text{of}\;\text{birth}\;\text{registration}=\frac{\text{Number}\;\text{of}\;\text{registered}\;\text{births}}{\text{Expected}\;\text{number}\;\text{of}\;\text{births}}\times 100.$

‡ Expected number of infant deaths=Number of registered infant deaths×IMR/1,000 (and IMR is assumed to be 43 for the state of Uttar Pradesh, based on SRS data^12^).

§ $\text{Estimated}\;\text{completeness}\;\text{of}\;\text{infant}\;\text{death}\;\text{registration}=\frac{\text{Number}\;\text{of}\;\text{registered}\;\text{infant}\;\text{deaths}}{\text{Expected}\;\text{number}\;\text{of}\;\text{infant}\;\text{deaths}}\times 100.$

The CGPP management team periodically performs desk reviews and physical verification (using an LQAS or household cluster-sampling method) to assess the data accuracy. The main parameters of data verification include SIA or RI coverage, whether SBCC field activities were performed, and perceptions of caregivers.

During physical verification, an investigator personally visits mothers/caregivers and verifies with her whether the data in the CB-MIS (e.g., the vaccination status of her children) as reported by CMCs or BMCs are accurate. The observed variation between reported and verified data helps to assess the accuracy and completeness of the CB-MIS.

Table 5 presents vaccination coverage and data accuracy observed during CGPP India’s data validation exercise in 2014. This exercise found a decent quality of reporting for OPV vaccination (OPV1, OPV2, and OPV3) among children aged 12–23 months. However, the entries of some of the vaccinations, particularly of hepatitis B, were not recorded/updated on the vaccination cards,‡ which was one of the main reasons for overreporting in RI coverage. This exercise helped CGPP India in identifying and improving the quality of data reporting. Immediately after this exercise, the CGPP established data validation processes as part of the routine monitoring and supervision mechanism and also periodically (at least once in a year) began to conduct systematic assessments to check and ensure data accuracy.

**Table 5:** Routine immunization coverage and data accuracy of the CORE Group Polio Project’s census-based management information system, 2014

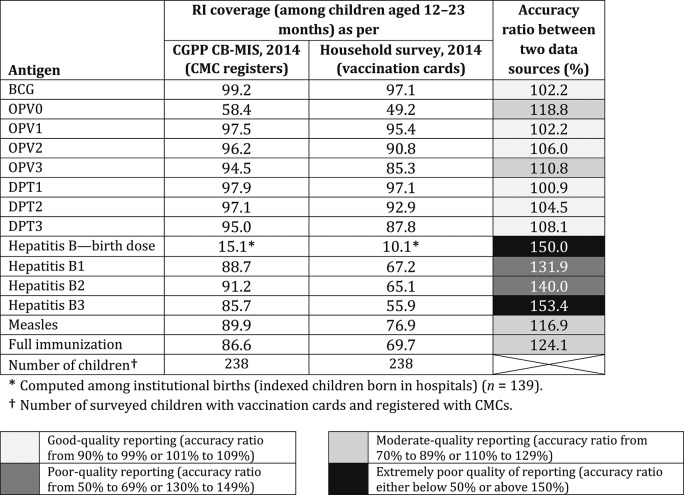

* Number of surveyed children with vaccination cards and registered with CMCs.

† Computed among institutional births (indexed children born in hospitals) (*n* = 139).

## DISCUSSION

Following the introduction of the CB-MIS in the CGPP catchment areas composed of hard-to-reach and resistant populations, there has been an increase in polio campaign coverage, a reduction in the percentage of households that resist polio immunization, and a sustained high level of RI coverage for polio and other antigens. The GoI’s priority on immunization and polio has been a major enabler for expanding vaccination coverage along with two decades of uninterrupted donor funding that has enabled the CGPP to continue its initiatives. Vaccination programs of low- and middle-income countries face a challenge of about 32% prevalence of missed opportunities for immunization (MOI).^23^ The CGPP CB-MIS helps in reducing the MOI through the inclusion of provider prompts (such as defaulter tracking and due-list for vaccination) and client prompts (e.g., RI invitation slips). Based on our experience, we consider that the following strengths of the CB-MIS have contributed to these achievements:

1. The CB-MIS makes it possible for the CMCs to create micro-plans based on solid household-level information at the community level.
2. Complete information is available for each child identified by household, enabling CMCs to implement CGPP interventions better.
3. The dual recordkeeping system of information compiled at CMC and BMC levels enables the use of information for both interventions at the household/community level (using disaggregated data) and management at the block level and higher (using aggregated data). Duplicate carbon copies of the monthly performance reports, which provide basic data such as number of live births and number of children vaccinated, are shared by CMCs/BMCs with their immediate supervisors. These data are not only used for reporting but are also used by the supervisors for validation.
4. The information is collected, compiled, and analyzed by the frontline workers (CMCs and BMCs) with minimum technical support, empowering them to improve the effectiveness of their own work.
5. The standardized templates for data compilation and analysis facilitate timely feedback to frontline workers and enable them to use this information for planning and implementation at their level.

For a health program that aims at universal coverage, information about every household and its participation in the program enables the program to determine which households are not participating, and then enables the program to target efforts on those households. In our case, the CB-MIS also provides the denominator for calculating the coverage indicators at each level of programming (CMC area, BMC area, and above), which is crucial for monitoring the service coverage of the target population and for identifying households and individuals that are not covered at any given point of time. The timely aggregation of data at the local and higher levels, such as at the block and district levels, is useful for middle-level managers. For a behavior-specific communication program, it is necessary to collect information on the households where the desired behavior is not occurring and determine the underlying reasons. This capacity is not possible for an MIS that relies on sampled households.

The CGPP in India has demonstrated that CMCs can make behavioral diagnoses and deal with negative behavior if they are provided with simple protocols and the necessary skills to use them. The CGPP has also demonstrated that there is potential and scope for monthly micro-planning for SBCC activities in the same way that the CB-MIS is used for micro-planning of immunization activities. The CGPP has demonstrated that its CB-MIS can be scaled up to cover a large population (3 million people). The CB-MIS has helped the CGPP in meticulous planning, execution, and monitoring of its intervention, applying the same approach for both the rural and urban areas. Frequent in- and out-migration in urban settings was a major issue that had to be dealt with by periodic updates of household information. Also, a specific mechanism had to be established to identify and track the children from areas with high rates of seasonal migration, such as those in nomadic families and those living at construction sites and brick kilns. The CB-MIS, originally designed for CGPP India, has been replicated in other CGPP countries. For instance, the CGPP in Nigeria has adopted the child tracking system to effectively execute polio campaigns and RI services. It could be adopted for other public health programs where case detection and follow-up are necessary, such as child survival programs or tuberculosis programs.

CMCs appointed by the CGPP have the same educational qualifications and sociodemographic characteristics as accredited social health activists (ASHAs). There are over 870,000 ASHAs^24^ appointed by the National Health Mission (NHM) in India who could benefit from the incorporation into their work of a CB-MIS similar to that developed by the CGPP. Accredited social health activists are the link between the community and service providers of the NHM. They assess the health needs of the community and maintain a record of health information for the assigned community in a Village Health and Index Register (VHIR). The VHIR is designed for ASHAs to register and track pregnancy, pregnancy outcomes, newborns, child vaccination, and other services that are offered by ASHAs. Accredited social health activists are also expected to maintain a record and report the vital events (births and deaths) in their areas to the authority responsible for maintaining the Civil Registration System.^25^ The existing system of vital events registration through ASHAs can be further strengthened by building the capacities of ASHA facilitators (supervisors of ASHAs) in monitoring and supportive supervision for maintenance and sharing of vital events with the Civil Registration System.

The CGPP CB-MIS includes most of the programmatic indicators needed for monitoring program inputs, processes, outputs, and outcomes, and it has been crucial in the planning, implementation, monitoring, and evaluation of the interventions of the CGPP. The CB-MIS has been essential for achieving near-universal polio coverage of children in hard-to-reach and resistant populations because it provides the means for assessing the progress of the program on a monthly basis and for identifying priority geographic areas where intensified efforts are needed.

Census-based management information systems are not widely used, unfortunately, although their potential has been recognized since the 1970s and even before to guide the use of scarce program resources to optimize benefits for population health, including the detection of infectious disease outbreaks.^22^ In the 1980s and 1990s, similar approaches were developed for strengthening census-based programming for reducing child mortality in Pakistan and Bolivia.^26–28^ More recently, a census-based MIS has been implemented in the slums of Freetown, Sierra Leone, as a means for engaging communities in the use of local data to improve their local health services and health outcomes.^29^ However, more experience with and rigorous evaluation of these CB-MISs are needed.

The CGPP’s CB-MIS has evolved over time from one with only several indicators to a full-fledged system with many indicators built into a log frame. The CGPP log frame provides a summary of interventions, expected results, and a monitoring and evaluation plan for each of the project’s objectives. Initially, the system used loose sheets of paper instead of uniform registers. Currently, the system uses standardized, printed registers.^30^ Initially, CMCs felt that it would be difficult to collect, compile, and analyze all the information required. However, CMCs now find the system to be manageable and can maintain their registers with minimal errors. The CGPP management team conducts capacity-building activities for CMCs, BMCs, and supervisors. These activities improve the ability of the field staff to accurately record and report information in a timely fashion and, ultimately, help the CGPP achieve its goals.

### How the CB-MIS contributes to program effectiveness.

**Figure 6.**
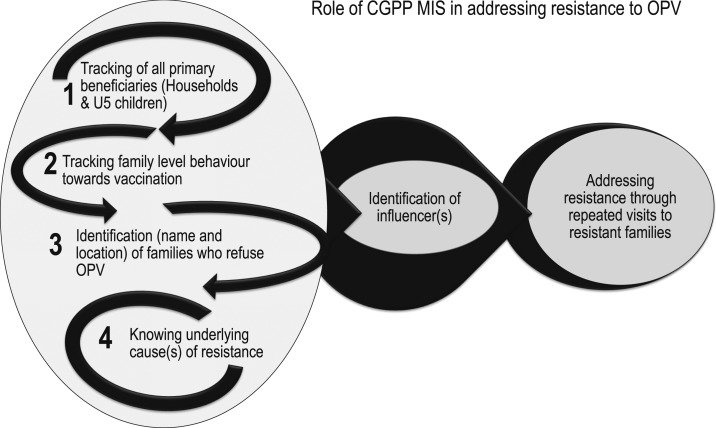
Depiction of the census-based management information system’s contribution in addressing resistance to polio immunization.

Social and behavior change communication activities reliant on epidemiological and social science data are crucial for the polio eradication program because it identifies every eligible child and the vaccination history of every eligible child. A unique aspect of CGPP strategies is the efficient utilization of the CB-MIS in the implementation of SBCC strategies. As the CGPP in India mainly supports polio and RI activities, the CB-MIS contributes to the design and implementation of SBCC activities for increasing immunization coverage at the time of polio SIAs and RI sessions. The same CB-MIS could be readily adapted to support programs addressed at other public health priorities. Figure 6 describes how the CB-MIS contributes to the tailoring of SBCC interventions to overcome resistance to OPV immunization. The CB-MIS generates real-time information on the following six key aspects.

#### Potential recipients of vaccination.

The CB-MIS helps CMCs track all primary beneficiaries (children younger than 5 years) for participation in SIAs and in RI sessions. The CMCs mark the location of every eligible child on their area maps. Before every RI session, they prepare a “due-list,” specifying the name of each child and the type of immunization needed.

#### Behavior of each family.

The vaccination-related behavior of each household in the CGPP catchment area is tracked by specifying the SIA vaccination status of each child. During the SIAs, CMCs accompany vaccinator teams and record the vaccination status of all children younger than 5 years in each house. At the time of each SIA, all the houses of the CMC catchment areas are marked with either a “P” or an “X,” and houses that are converted from “X” to “P” are marked as “X-P.” This house-marking is universally used for polio SIAs in the entire state of Uttar Pradesh, and CMC registers follow a similar marking system. The designation “P” means either 1) all the children received a polio immunization at the time of that SIA or 2) there are no children in the household. The “X” designation signifies that either 1) children are present who were missed during the SIA or 2) the SIA vaccination status of the children is not known. The CMC registers further classify “X” houses into the following six categories:

1. “XR” houses—families who refuse an SIA vaccination
2. “XL” houses—locked houses with no likely possibility of families returning within 6 weeks (so marked after checking with neighbors)
3. “XH” houses—houses with children who were away from home at the time of house visit of vaccinators
4. “XS” houses—houses with sick children
5. “XV” houses—houses with children who are “out of station” (houses with children who are not expected to return home before the end of the SIA)
6. Houses that are converted from “X” to “P” are marked as “X-P” on the houses and in registers.

Although there was some opposition to marking homes at the outset, families generally appreciate the importance of this system, and few families remove the mark. The same information is also maintained in the CMC record.

#### Location of resistant families.

The CMCs identify households that have the potential for resistance or are known to be resistant to having their child vaccinated against polio during SIAs and at the time of RI sessions. The potentially resistant households may include families with a history of an adverse event following immunization, families belonging to an area where a severe adverse event was reported, or families from an area where an anti-vaccination group has actively disseminated messages in the form of *fatwa.*§

#### Underlying cause(s) of resistance.

Community mobilization coordinators investigate the reason(s) behind the refusal by having personal interactions during home visits to each resistant family.

#### Identification of appropriate influencers.

The CB-MIS maintains a CMC area-wide list of potential local influencers who are recognized and respected in the community, such as village heads, religious leaders, doctors, ration dealers (who are owners of “fair price shops,” which are part of the government’s public distribution system to sell or distribute subsidized food and other items), and schoolteachers.

#### Counseling of resistant families.

Having identified resistant or potentially resistant families and appropriate influencers, CGPP field staff (mainly CMCs and BMCs, but sometimes the district mobilization coordinator as well) and influencers visit the families repeatedly, if necessary.

The CB-MIS supports program implementation teams in the effective management (planning, implementation, supervision, monitoring, and evaluation) of SBCC interventions at various operational levels (from the household to the community level to the block level and above). The capacity-building efforts of the CGPP’s management team are a driving force behind the success of the CB-MIS in supporting the interventions. Periodic training and on-the-ground support by supervisors not only build confidence among the field staff but also improve the completeness and accuracy of recording and reporting.

Although the CB-MIS was not primarily designed for the registration of vital events, it performs better than the government’s Civil Registration System in Uttar Pradesh. We estimate that the CGPP CB-MIS registered 85% of births and 98% of infant deaths. These results demonstrate the feasibility of the CB-MIS for guiding local program activities and improving the vital events registry. CORE Group Polio Project CMCs along with other frontline workers could have a formal link with the Civil Registration System to register the events there and at the same time generate awareness about the value of birth and death registration. The CGPP CMCs encourage communities to register all births with the civil authorities.

A CB-MIS primarily used for the management of a specific program can also be helpful in strengthening the vital events registry. Vital statistics are important indicators of health status, and they are essential for effective planning and implementation of policies and services at the population level.^31,32^ A robust civil registration and vital statistics system plays a critical role in addressing health inequities and strengthening global health and development efforts.^31^ Vital events registration systems vary by country and apply the principles of prospective and retrospective registration of vital events, such as births, deaths, and marriages.^14,31,33–36^ The lack of completeness of civil vital events registration systems is a concern in almost all low- and middle-income countries.^32–34,36–38^ The civil registration and vital statistics systems are usually managed by governments to record every birth, adoption, death, marriage, and divorce that occurs among a country’s population.^31^

In India, the Civil Registration System and the SRS are the two major sources of vital statistics estimation.‖ Also, the National Family Health Survey (a demographic and health survey), carried out every 5–10 years, provides estimates of mortality of children younger than five years and maternal mortality from household sample surveys.^17^ The Civil Registration System in India registers vital events (births, deaths, and stillbirths) as the family members report them. The Civil Registration System should be the best source of information on vital events and the population-level rates of these events at all levels. It follows a unified process of recording of vital events that is supposed to be continuous, permanent, compulsory, and universal.^14^ Unfortunately, the birth and death registration rates in the Civil Registration System are quite low in most parts of India, a reflection of both supply-side and demand-side factors.^14,38^ Improvements in supply-side factors, such as overcoming political, administrative, economic, and legislative barriers,^38^ and improvements in demand-side factors, such as improving knowledge about and the value of registration in the general population, are needed to improve the completeness of vital events registration in India.

The report on *Vital Statistics of India, 2015* states that 88% of births nationally were registered through the Civil Registration System. However, a significant gap between the expected number of births and the actual number of registered births has been observed for some states in India, including Uttar Pradesh.^14^ According to our estimate, only 67% of births and 9% of infant deaths are recorded in the Civil Registration System in Uttar Pradesh. The potential of a strong CB-MIS with its own vital events registration component as part of a health or development program is not often recognized as a contributor to strengthening the Civil Registration System.

## CONCLUSION

The CB-MIS developed by the CGPP in India has enabled the CGPP to improve its performance in hard-to-reach and resistant populations at high risk for polio transmission. Registration of pregnancies and births makes it possible to track all young children, target newborns for administration of the birth dose for polio immunization, and identify children in need of RIs. The CB-MIS vital events registration system appears to be almost as accurate as the “gold standard,” the GoI’s SRS. It also has the added advantage of being able to use the data to guide local management and field activities. This CB-MIS could be used for other purposes beyond polio eradication and RI, such as child survival and tuberculosis (TB) programming.

The case-tracking system identifies immunization defaulters and is helpful in delivering the case-specific personalized inputs required to improve program outcomes. Monthly aggregate reporting helps middle-level managers to monitor progress. The CMCs, who are local community-based health workers responsible for about 500 households, can make behavioral diagnoses if they are provided with simple protocols and the skills necessary to use them. The CGPP has been able to achieve a high level of birth and infant death registration in a population of three million people during the period from October 2014 to September 2017, thereby demonstrating the potential of the CB-MIS for strengthening vital events registration at scale. In conclusion, the CGPP CB-MIS has been instrumental for polio eradication and RI activities in high-risk areas of Uttar Pradesh where hard-to-reach and resistant groups are located. The CB-MIS could be adapted for use in improving the civil registration of vital events and in addressing other public health priorities in underserved populations with a high disease burden, including childhood survival and TB programs, for which case detection and follow-up are necessary.

## Supplemental appendix I

Supplemental materials

## Footnotes

† † In India, a block is the lowest governmental administrative unit and consists of approximately 200,000 people.

‡ ‡  The vaccination card is also known as an RI card or an MCP (mother and child protection) card. In India, children receive RI cards from the public (government) or from the private health delivery system. In the government health system, the public health-care provider (e.g., the auxiliary nurse midwife) provides MCP cards to pregnant women at the time of antenatal registration. The MCP card has a provision for recording the vaccination status and maternal health-care services (including tetanus toxoid immunization), whereas most of the RI cards provided by private institutions record immunizations only. Whenever a child is vaccinated, the vaccinator records the date of immunization on the card.

§ § A fatwa is an interpretation or opinion of Islamic religious law by a qualified cleric.

‖ ‖ The nationwide Civil Registration System is supposed to cover everyone. The SRS is implemented in a sample of representative catchment areas and uses an enumerator who visits every house every 6 months to record vital events.^22,24,25^
