# Supplementary material for: Effectiveness of a Census-Based Management Information System for Guiding Polio Eradication and Routine Immunization Activities: Evidence from the CORE Group Polio Project in Uttar Pradesh, India
Source: Am J Trop Med Hyg. 2019 Oct;101(4 Suppl):33–44. doi: 10.4269/ajtmh.18-0935 (PMC6776090; doi:10.4269/ajtmh.18-0935)
Supplement: Supplementary file 1 [file tpmd180935.SD1.docx]

**Supplemental APPENDIX**

**SUPPLEMENTAL APPENDIX I: Details of the CORE Group Polio Project’s Social and Behavior Change Communication Activities for Polio Supplemental Immunization Activities and Its Activities for Routine Immunizations, Including Routine Polio Immunization**

**SBCC activities for polio SIAs.** During the SIAs, CMCs assist vaccinators by setting up vaccination booths, organizing groups of child mobilizers (*Bulawwa tollies*), and arranging for mosque and temple announcements. The CMCs also accompany vaccinator teams to all the houses and work to convince families with an unvaccinated child (called an “X” household) to allow the child to be vaccinated (converting an “X” household to a “P” household, denoting a house where all eligible children are vaccinated against polio). Lastly, the CMCs accompany persons of influence such as community and religious leaders during follow-up visits to resistant households.^21^ In between the SIAs, the CMCs carry out activities aimed at increasing the coverage of OPV (and of other antigens of RI) by visiting houses and talking to mothers, other caregivers, as well as fathers and elderly members of the household in order to dispel their doubts or combat rumors about immunization. The CMCs also hold meetings with mothers to discuss broader health issues such as children’s health problems along with the prevention and management of common illnesses.^11, 20, 39^

**CGPP activities for routine immunization (including routine polio immunization).** The CGPP in India implements a variety of SBCC interventions and applies the following approaches for strengthening RI:

1. Tracking of pregnant women, newborns, and eligible children
2. Ensuring the availability of immunization cards to every child and recording vaccination status on that card
3. Preparing a list of all children who need a vaccination prior to every RI session and distributing invitation slips to caregivers that inform them of the vaccination(s) due as well as the time and venue of the RI session site. Caregivers of children in need of an immunization bring RI invitation slips to RI sessions and the vaccinators (and CGPP mobilizers) maintain a record of all the vaccinated children. At the RI session site, CGPP mobilizers (CMCs) also mark the vaccination status (vaccinated or not-vaccinated) against every child on the due-list to ensure the vaccination of all invited children.
4. Targeting inter-personal communication visits to the families who previously refused vaccinations
5. Holding group meetings with community members to dispel myths and misconceptions about immunizations
6. Assisting in preparation of RI micro-plans with the local immunization team and to ensure availability of vaccines, vaccinators, and other logistics
7. Assisting in the monitoring of RI sessions to ensure quality
8. Ensuring delivery of four key messages to caregivers: (i) What vaccine is given and what disease it prevents, (ii) When to come for the next immunization, (iii) What minor adverse events could occur and how to deal with them, and (iv) The importance of keeping the immunization card safe and bringing it to the next visit.
9. Conducting timely follow-up visits to recently vaccinated children and counseling caregivers in case of an adverse event following immunization
10. Performing periodic assessments of coverage and factors affecting immunization coverage.

**SUPPLEMENTAL APPENDIX II. DETAILS OF THE CENSUS-BASED MANAGEMENT INFORMATION SYSTEM**

Community Mobilization Coordinators (CMCs) maintain the Child Tracking Register. It is the most basic component of the CB-MIS. It contains information about the following:

- Socio-demographic characteristics of the CMC work area, including names and contact details of influential people
- Information about community resources such as schools, *madrasas*, mosques, and temples
- Supplementary Immunization Activity (SIA) campaign-specific vaccination status of all households (Appendix Figure 1)
- Tracking of pregnant women and newborns (identification of pregnant women and more frequent targeted visits from CMCs ’s visits to pregnant women and pregnancy outcome), and,
- Routine immunization history for each child younger than 5 years of age (Appendix Figure 2).

The household- and child-specific information of the Child Tracking Register provides information on each household and on each child, making it possible to carry out micro- planning for detailed implementation. More importantly, this Register provides the denominators by which monthly monitoring of the program at the level of each CMC is undertaken. Using denominators such as “number of children eligible for routine immunization,” CMCs compute by hand the vaccination coverage of their catchment areas. After identifying areas with low routine immunization (RI) coverage or with high levels of RI defaulters, CGPP supervisors take remedial actions.


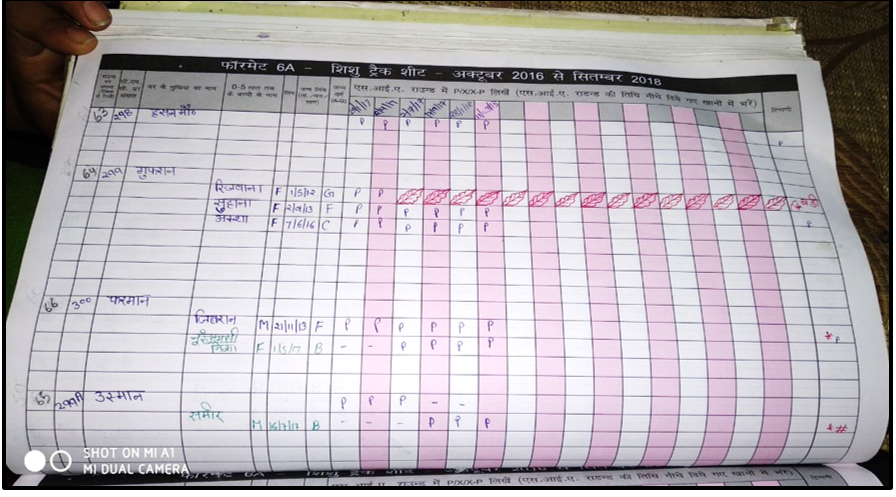


Format 6A – Child Tracking Sheet – October 2018 to September 2018

***Column headings:*** Col. 1) House number, Col. 2) CMC house number, Col. 3) Name of household head, Col. 4) Name(s) of under-five-year child, Col. 5) Sex of child, Col. 6) Date of birth (DD/MM/YY), Col. 7) Birth year (A-G), Cols..8 to 24) SIA-specific vaccination status of the household (P or X or X-P), Col. 25) Remarks

**Supplemental Appendix Figure 1. Household information from the Child Tracking Register used for planning Supplemental Immunization Activities**


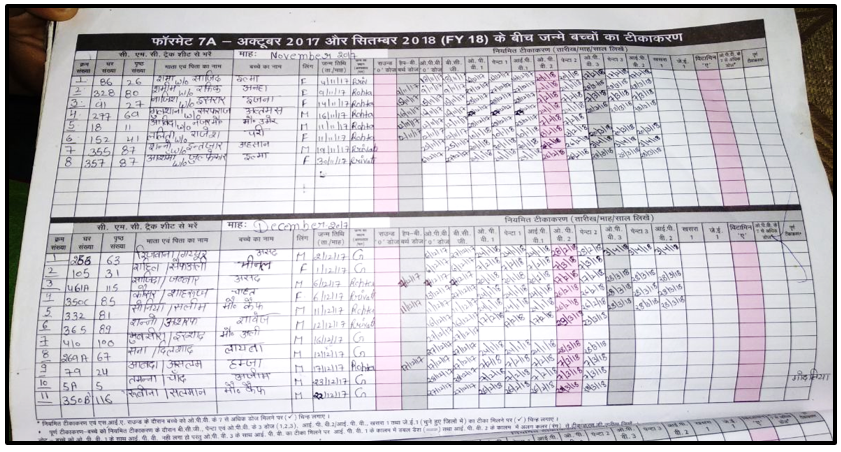


Format 7A – Vaccination status of children born from October 2017 to September 2018

***Column headings:*** Col. 1) House number, Col. 2) House number., Col. 3) Page Number 4) Name of parents (father and mother), Col. 5) Name of child, Col. 6) Sex of child, Col. 7) Date of birth (DD/MM/YY), Col. 8) Birth year (A-G), Col. 9) Vaccination date, Col 9) SIA OPV0, Col. 10) Hepatitis B birth dose, Col. 11) OPV0, Col. 12) BCG, Col 13) OPV 1, Col. 14) Penta 1, Col. 15) fIPV1 Col. 21) Measles 1, Col. 22, JE 1, Col. 23) Vitamin A, Col. 24) Received 8+ OPV dose – Yes/No, Col. 25) Fully immunized – Yes/No

**Supplemental Appendix Figure 2. Routine immunization information for specific children in the Child Tracking Register**

The Supplemental Immunization Activities (SIA) register provides aggregated information about the coverage of the SIA campaigns (through the polio booths and house-to-house vaccination) and a list of missed children from all campaigns (Appendix Figure 3). The SIA register is the key MIS for addressing resistance to polio vaccination. It is a detailed information system demonstrating the magnitude of the problems of non-vaccination or resistance and their underlying causes. It also provides information on the extent to which the problems are addressed in each CMC’s location.


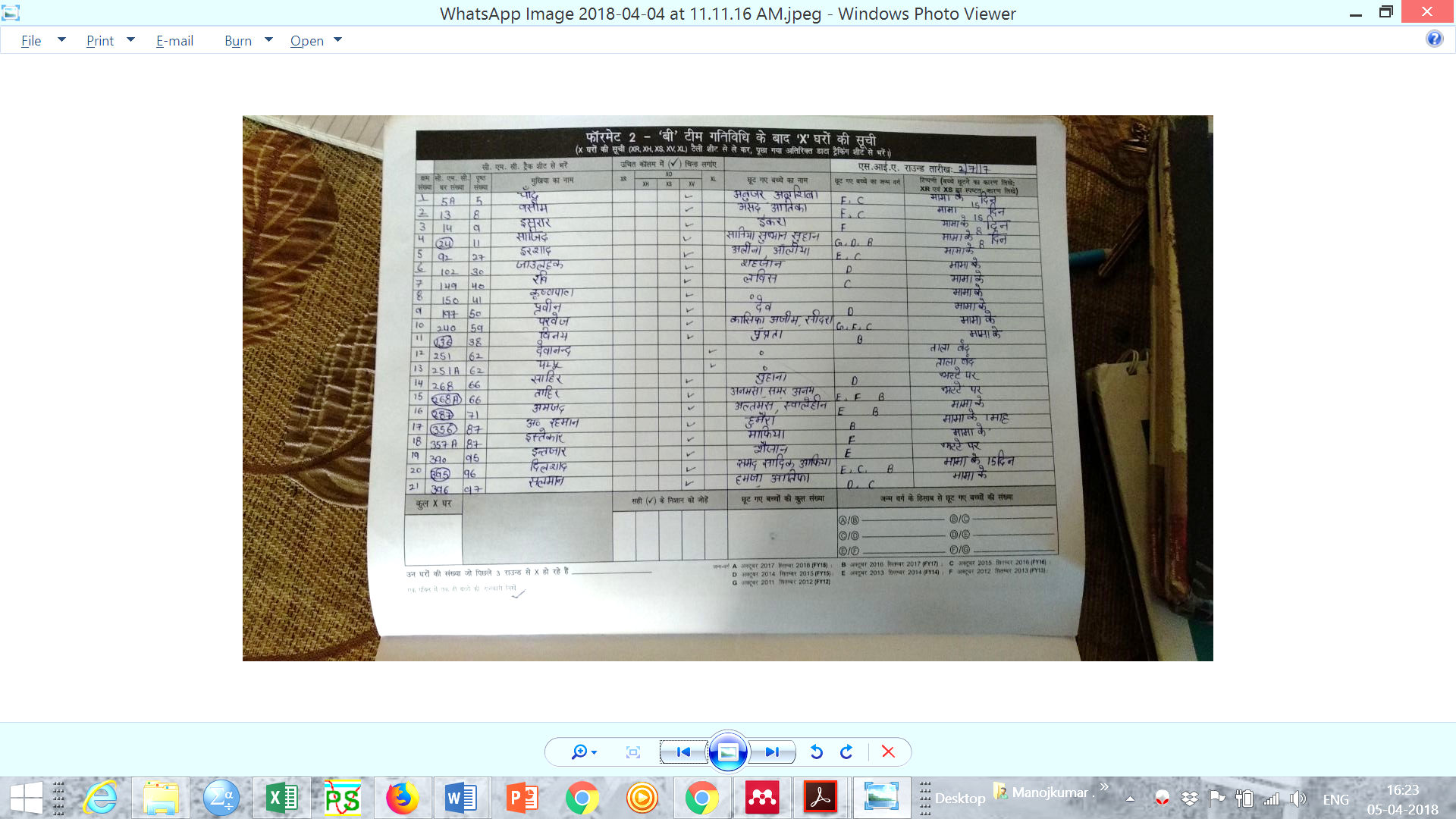


Format 2 – List of remaining ‘X’ houses after ‘B’ team activity

***Column headings:*** Col. 1) House number, Col. 2) CMC house number Col. 3) Page number Col. 4) Name of household head, Cols. 5-9) Category of ‘X’ houses: Col. 5) XR houses, Col. 6) XH houses, Col. 7) XS houses, Col. 8) XV houses, Col. 9) XL houses, Col. 10), Name of missed child/children, Col. 11) Age group of missed children (A/B/C/D/E/E/G)), Col. 12) Remarks (specify reasons behind children missing SIA vaccination)

**Supplemental Appendix Figure 3. Supplemental Immunization Activity Register with list of missed houses**

**and missed children**

**
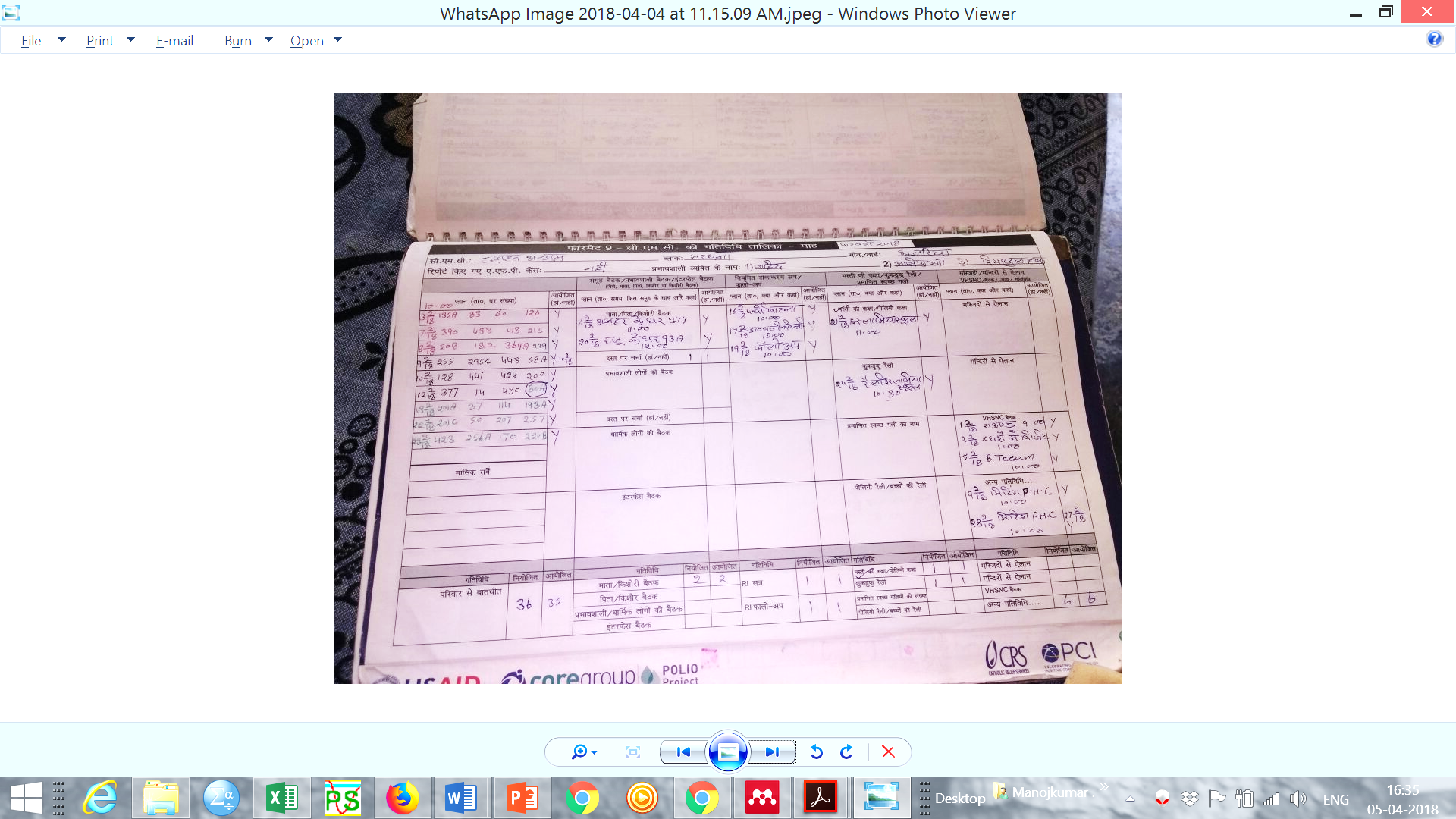
**

Format 9 – CMC monthly planning sheet

***Column headings:*** Col. 1) Plan, Col. 2) Execution of plan (Held: Yes/No, Col. 2) IPC contacts (home visits), Col. 3) Plan of group meetings/influencers meetings and Col. 4) Execution of plan (Held: Yes/No, Col. 5) Plan of RI session/follow-up and Col. 6) Execution of plan (Held: Yes/No, Col. 7) Plan of child- centric activities and Col. 8) Execution (Held: Yes/No, Col. 9), Plan of mosque/temple announcements/VHSNC meetings/Other activities and Col. 10) Execution (Held: Yes/No, col. 10)

**Supplemental Appendix Figure 4. A Community Mobilization Coordinator Register showing sheet**

**for monthly planning**

The Planning and Reporting Register uses information from the Child Tracking Registers for planning monthly activities of the CMCs. Information for the input and output indicators comes from these registers (Appendix Figure 4). The CMCs aggregate information from the Child Tracking Registers to provide details for the Planning and Reporting Register of planned versus actual activities, utilization, and polio immunization coverage. These registers are useful for the program staff involved in the monthly monitoring of inputs and outputs.

CMCs prepare maps of their catchment areas that depict key locations and all houses (see Appendix Figure 5). The Planning and Reporting Registers of the CMCs and BMCs provide dual record keeping. The CMCs share a carbon copy of aggregated data with their respective supervisors (the BMCs), and the original data sheet remains in the CMC registers for further reference. The aggregated information is further compiled for each block in the BMC register.


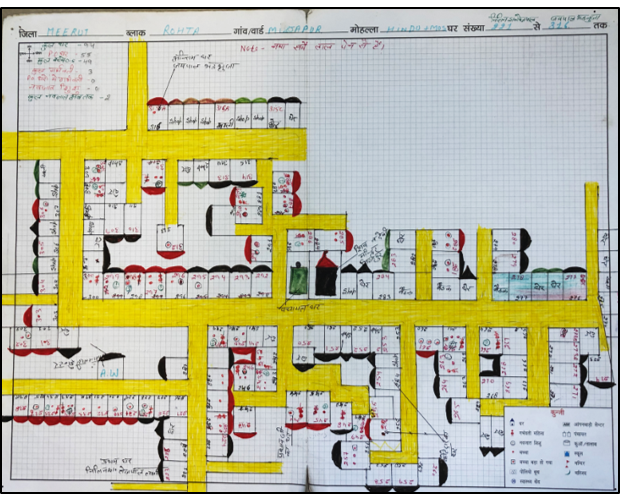


**Supplemental Appendix Figure 5. An example of a map of a catchment area of a Community Mobilization Coordinator**


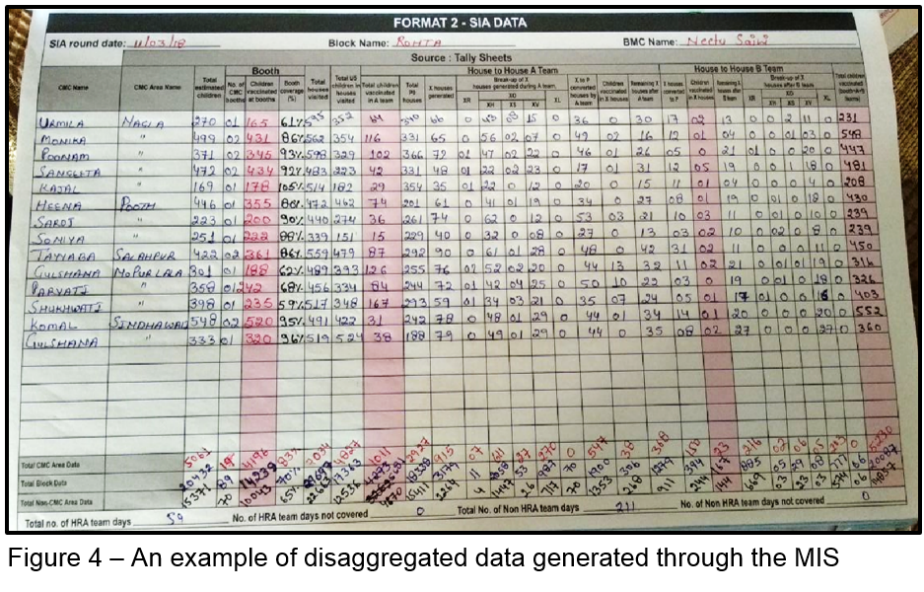


**Supplemental Appendix Figure 6. A Block Mobilization Coordinator’s Register with aggregated data**

**from Community Mobilization Coordinators**

BMCs maintain information about their respective areas in two registers: (1) BMC register and (2) High-risk group (HRG) register. A BMC register contains the following information:

- A profile of the BMC work area – includes the number of villages/urban areas, high- risk areas in the block, and the number of areas covered by CMCs
- A profile of CMCs – includes information about their age, sex, education, marital status, religion, date of joining CGPP, number of houses covered, as well as number of under-five and under-15 children
- Aggregated information from CMCs on Supplementary Immunization Activity (see Appendix Figure 6)
- Aggregated information from CMCs on number of RI sessions held
- Aggregated information from CMCs on births, child deaths, and RI coverage
- Planning and reporting sheet for BMC activities
- Aggregated information regarding activities performed by CMCs
- Information on acute flaccid paralysis (AFP) cases reported from the BMC work area
- Summary of information on high-risk groups (HRGs) - number of HRG sites, number of families and under-five children in HRGs, SBCC activities at the HRG sites, and vaccination status of children in HRGs

An HRG register maintained by the BMC includes following information:

- Information on date of arrival and expected date of departure of families from HRGs, number of families, number of under-five children, and number of children receiving RI against expected
- List of informants who provide information about the arrival or departure of HRGs in BMC work area
- Tracking sheet for vaccination status of under-five children during SIAs and RIs

The project MIS uses MS Excel-based standardized templates for the entry and analysis of aggregated data at the district level and groups of selected districts (called sub-regions).
